# Differences in COVID-19 Vaccine Concerns Among Asian Americans and Pacific Islanders: The COMPASS Survey

**DOI:** 10.1007/s40615-021-01037-0

**Published:** 2021-04-14

**Authors:** Van Ta Park, Marcelle Dougan, Oanh Meyer, Bora Nam, Marian Tzuang, Linda Park, Quyen Vuong, Janice Tsoh

**Affiliations:** 1 School of Nursing, Department of Community Health Systems, University of California, San Francisco (UCSF), San Francisco, CA USA; 2 Asian American Research Center on Health (ARCH), UCSF, San Francisco, CA USA; 3 Department of Public Health and Recreation, San Jose State University, San Jose, CA USA; 4 Department of Neurology, University of California, Davis (UCD), Davis, CA USA; 5 International Children Assistance Network, Milpitas, CA USA; 6 School of Medicine, Department of Psychiatry and Behavioral Sciences, UCSF, San Francisco, CA USA

**Keywords:** COVID-19, vaccine concerns, Asian Americans, Pacific Islanders

## Abstract

**Background:**

Understanding concerns for receiving COVID-19 vaccines is key to ensuring appropriately tailored health communications to increase vaccine uptake. However, limited data exists about vaccine concerns among Asian Americans and Pacific Islanders (AAPI).

**Methods:**

Data from the **C**OVID-19 Effects on the Mental and Physical Health of AAPI Survey Study (COMPASS), a cross-sectional, national survey for AAPI adults in the U.S. were used (N=1,646). Descriptive statistics were used to assess sample characteristics including proportions of AAPI with various COVID-19 vaccine concerns, categorized as *none*, *side-effects only, unsafe only, and multiple reasons,* and differences in vaccine concerns by socio-demographics. Ordinary multivariable logistic regression analyses were conducted to evaluate associations between a characteristic and having *any* vaccine concerns.

**Results:**

Overall, 76% of the respondents reported having at ≥1 concerns about the vaccine. The most common concern was side effects (65%). Vietnamese Americans reported less concerns (vs. Chinese Americans). Those who were 30-39 and 40-49 years old (vs. <30), females (vs. males), and experienced mild negative impacts from COVID-19 on family income/employment (vs. no change) reported more concerns about the vaccine. Those who had less vaccine concerns were those who reported higher (vs. low) health status, ≥60 years old (vs. <30), and separated/divorced/widowed (vs. single).

**Discussion:**

AAPI is a diverse population and this study revealed differences in vaccine concerns across AAPI groups. Findings revealed potential targets for patient education needs. Effective strategies to address various vaccine concerns across subgroups of AAPI will be crucial to ensure equity in vaccination uptake.

## Introduction

Addressing concerns regarding coronavirus disease 2019 (COVID-19) vaccination is a critical issue affecting the success of vaccination programs and bringing much needed control to the pandemic. Although a recent survey [1] found an increase in the proportion of US adults with greater degree of vaccine acceptance compared to early months of the pandemic [2, 3], a sizeable proportion of the US population is still unsure or do not plan to become vaccinated against COVID-19 [4]. The accelerated pace of vaccine development and the politicization of vaccine approval has heightened public anxieties and could have influenced its acceptance [5, 6].

It is essential that public health messages promoting COVID-19 vaccination target the underlying concerns especially for individuals who are among the vaccine “wait and see” group. This “wait and see group” is particularly important, given that they may be easier to convert from ambivalent towards the vaccine to being willing to get it, compared to those who outright rejected to receive the vaccine. A recent poll by the Kaiser Family Foundation survey reported that 31% of the individuals surveyed belong to this group, saying that they will wait until the vaccine is available for a while and see how it is working for others who have taken it [4]. Several US studies have reported on specific concerns regarding the uptake of the COVID-19 vaccine including vaccine safety (i.e., side effects) [1–3, 7–9], vaccine effectiveness (including the need for more information about the vaccine) [2, 7], anti-vaccine beliefs and attitudes [1, 3], being worried about getting COVID-19 from the vaccine [1, 9], lack of trust [3], and perceived lack of testing [7].

A major gap in our understanding of concerns regarding COVID-19 vaccine is the scarcity of Asian Americans and Pacific Islander (AAPI) participation and thus their perspectives in these studies. To the authors’ knowledge, only two polls have surveyed Asian Americans about vaccine willingness, but the number of survey responses was few, and the respondents were limited to Asian Americans who could complete the surveys in English [10, 11]. Moreover, neither of the surveys were able to provide disaggregated AAPI data, which runs the risk of masking possible disparities across AAPI subgroups.

This study aimed to take a deeper dive to understand the different types of concerns related to COVID-19 vaccination and whether such concerns differ by characteristics between and within subgroups of the AAPI population using a large, ongoing multilingual survey called the **C**OVID-19 Effects on the Mental and Physical Health of AAPI Survey Study (COMPASS). The authors recognize that surveys such as this one are conducted in the context of a highly dynamic and changing landscape. However, given growing reports of inequalities in COVID-19 infection and mortality among AAPI [12–14], it is important to shed light on COVID-19 vaccine concerns specific to AAPI, especially when these may become barriers and/or facilitators to vaccination efforts that lead to achievement of herd immunity against COVID-19.

## Methods

### Study Eligibility, Recruitment, and Procedures

COMPASS is a cross-sectional, community-based national survey that assesses the COVID-19 effects on AAPI. To be eligible, participants must self-identify as AAPI alone or in combination with other races/ethnicities; be able to read English/Chinese (traditional or simplified Chinese)/Korean/Vietnamese; be ≥ 18 years old; and reside in the USA. The survey is available online (https://compass.ucsf.edu/) with as needed survey administration assistance by phone in the above-mentioned languages. COMPASS is an ongoing survey study, and this paper reports on 1646 participants who completed the survey from October 24–December 11, 2020, which was selected as the cutoff date for this analysis since it was the first day that the FDA authorized a COVID-19 vaccine [15]. The mean survey completion time was 21.6 (standard deviation (SD) = 15.5) min. Each participant was provided an option of receiving a $10 gift card upon survey completion.

Participants heard about COMPASS through community partners who serve AAPI, personal/professional networks, social media, email/listservs, flyers, and ethnic media. COMPASS also leveraged the Collaborative Approach for AAPI Research and Education (CARE) Registry [16] to recruit participants by email announcements. After providing e-informed consent (*n* = 1535) or verbal consent via phone (*n* = 111), 1646 participants completed the COVID-19 survey. The survey used Research Electronic Data Capture (REDCap) tools hosted at the University of California, San Francisco (UCSF) [17, 18].

### Measures

#### Dependent Variable

*COVID-19 Vaccine Concerns* [19] were assessed with the following question: “Some people may have concerns about COVID-19 vaccines, do you have any of the concerns below (please check all that apply)?” Response options included: (1) I do not have any concerns; (2) I’m concerned about potential side effects; (3) I think COVID-19 vaccine may not be safe; (4) I do not think that COVID-19 is dangerous to my health; (5) I am against vaccination in general; (6) The best way is for nature to take its course; (7) I believe natural or traditional remedies; (8) I’m afraid of injections; (9) Religious reasons; and (10) Others (please describe). The survey also elicited vaccine concerns via open-ended questions.

#### Independent Variables

*Socio-demographic* items were drawn from CARE [16]. Variables included race, cultural group, sex, sexual orientation, year of birth, nativity, years lived in the USA, marital status, employment, education, and annual household income in 2019. Participants were asked how well they could speak/read/write English.

Participants completed several existing surveys related to COVID-19 including the *Coronavirus Impact Scale (CIS)* related to changes in family income/employment [20] and *COVID-19 status* [21] (yes, no, unsure diagnosis).

Participants answered duration of *Shelter-in-Place (SIP)* questions based on *region*, per the Census Bureau’s definition of region (Midwest/Northeast/South/West) [22] which was obtained by converting the zip code, or Internet protocol address in the case of missing zip codes (*n* = 188). *SIP and Perceived Severity of COVID-19* items were developed by COMPASS.

*General health* was measured by asking participants to indicate their health “today” on a scale from 0 (worst) to 100 (the best health you can imagine) using the EQ-5D [23, 24] item, which was categorized into quintiles.

### Translation Process

The World Health Organization’s process of translation and adaptation of instruments [25] was used to guide the translations of the study materials that were not already available in the targeted language(s).

### Statistical Analysis

The outcome variable, vaccine concerns, was examined in two ways: (1) concerns (none/any), unsafe (yes/no), side effects (yes/no), and (2) number of concerns (none, one, 2 or more). Chi-squared tests were used to examine the association separately for unsafe, side effects, and number of concerns, and hypothesized factors associated with vaccine concerns, specifically race, cultural group, sex, sexual orientation, age, nativity, marital status, employment, education, household income, length of shelter in place, perceived severity of COVID-19, effect of coronavirus on family income, region, and general health status. Participants who responded that they could speak/read/write English less than very well (“some,” “a little bit,” or “not at all”) were categorized as having limited English proficiency (LEP) [26]. This study used binary logistic regressions to model the association between having vaccine concerns (none/any) and these same factors. Variables that attained a *p* value of < 0.10 in bivariate logistic regression analyses were included in the final model. All statistical tests were two-sided. Hosmer-Lemeshow goodness of fit test [27] indicated an acceptable fit of the final model (*P* = 0.18). Statistical analyses were conducted using SAS Software [28].

Open-ended responses were analyzed by a line-by-line reading of the data and then categorized into major codes and then themes with accompanying illustrative quotes. The analysis was done by one of the lead investigators with prior experience in qualitative analysis.

### Human Subject Protection

This study was approved by UCSF’s Institutional Review Board (protocol 20-31925).

## Results

### Sample Characteristics

Sample characteristics (*N* = 1646) are shown in Table 1. Participants included 97.6% Asian Americans and 2.4% Native Hawaiians and Pacific Islanders (NHPI). The major cultural groups included ethnic Chinese (including persons from Hong Kong and Taiwan; 37.1%), Vietnamese (29.0%), and Korean (20.5%). The sample comprised of more females (62.5%) and mostly heterosexuals (90.0%). The mean age of participants was 40.6 years (SD: 15.8) and ranged from 18 to 88. Overall, 61.5% of participants were foreign-born who had lived in the USA an average of 22.8 years (SD: 13.6), and 20% had limited English proficiency. Many completed the survey in English (73.3%).

**Table 1:** COMPASS study sample characteristics and COVID-19 vaccine concerns (*N* = 1646)

	N	%
COVID-19 vaccine concerns		
None	386	23.5
Side effects only	562	34.1
Unsafe only	99	6.0
Multiple reasons	599	36.4
Number of COVID-19 vaccine concerns		
None	391	23.8
One	723	43.9
Two or more	532	32.3
Race		
Asian	1607	97.6
Native Hawaiians and Pacific Islanders	39	2.4
Cultural group		
Asian Indian	28	1.7
Ethnic Chinese^1^	611	37.1
Filipino	71	4.3
Japanese	29	1.8
Korean	337	20.5
Native Hawaiian	17	1.0
Samoan	13	0.8
Vietnamese	477	29.0
Others/mixed	63	6.9
Sex		
Female	1,028	62.5
Male	601	36.5
Others/decline to state^2^	17	1.0
Sexual orientation		
Heterosexual	1,478	90.0
Not heterosexual	95	5.8
Decline to State	69	4.2
Age (in years)	40.6 (15.8)^3^; range: 18–88	
< 30	535	32.5
30–39	339	20.6
40–49	237	14.4
50–59	295	17.9
> 60	240	14.6
Nativity		
US-born	619	37.6
Foreign-born	1,012	61.5
Years in the USA	22.8 (13.6)^3^; range: 0–83	
Don’t know	15	0.9
Limited English proficiency (LEP)^4^		
Yes	335	20.4
No	1311	79.7
Marital status		
Single	564	34.3
Married/living with partner	988	60.0
Separated/divorced/widowed	83	5.0
Declined	11	0.7
Employment status		
Full-time	735	44.7
Part-time	287	17.4
Homemaker	150	9.1
Unemployed	213	12.9
Retired	141	8.6
Others/decline to state	120	7.3
Education		
High school or less	247	15.2
Some college or technical school	238	14.6
Bachelor’s degree	633	39.0
Master's degree or higher	507	31.2
Annual household income in 2019 ($)		
≤ 25,000	283	17.2
> 25,000–75,000	530	32.2
> 75,000–150,000	408	24.8
> 150,000	258	15.7
Decline to state	167	10.2
Tested positive for COVID-19		
Yes	33	2.0
No	1485	90.2
Not sure	108	6.7
Missing	20	1.2
Willing to get COVID-19 vaccine		
Definitely yes	725	44.0
Probably yes	503	30.6
Unsure	295	17.9
Probably no	82	5.0
No	41	2.5
Length of SIP ^5^ Order		
No order	93	5.7
< 1 month	111	6.8
1 to <2 months	227	13.8
2 to <3 months	223	13.6
3 months or longer	855	52.1
Do not know	132	8.0
The severity of COVID where you live		
A lot less	146	8.9
Somewhat less	338	20.6
About the same	417	25.4
Somewhat more	502	30.6
A lot more	236	14.4
COVID-19 effect on family income/employment		
No change	587	35.9
Mild	554	33.9
Moderate	439	26.8
Severe	56	3.4
Census region		
Midwest	90	5.8
Northeast	142	8.6
South	305	18.5
West	1109	67.4
Self-reported health, quintiles (range of health score)	78.2 (15.7)^3^; range: 1–100	
Q1 (1–70)	345	22.2
Q2 (71–78)	264	17.0
Q3 (79–83)	324	20.8
Q4 (84–90)	351	22.6
Q5 (91–100)	270	17.4

^1^Ethnic Chinese includes mainland Chinese, Hongkonger, Taiwanese, and Huaren

^2^Others: *n* = 7; decline: *n* = 6

^3^Mean (SD)

^4^Self-rated English proficiency categorized as limited if speaking or reading or writing English indicated as “some,” “a little,” or “not at all”

^5^*SIP* shelter-in-place

### COVID-19 Impacts

More than 64% reported that the COVID-19 had impacted their family income and employment (Table 1). Over half (52.1%) indicated their SIP was 3 months or longer; 45.0% perceived the COVID-19 severity of where they lived was “somewhat” to “a lot” more than other US areas. Approximately, two-thirds of participants reported mild to severe impact on family income and employment due to the COVID-19. Few (2.0%) said they had tested positive for COVID-19.

### COVID-19 Vaccine Concerns and Willingness

**Fig. 1:**
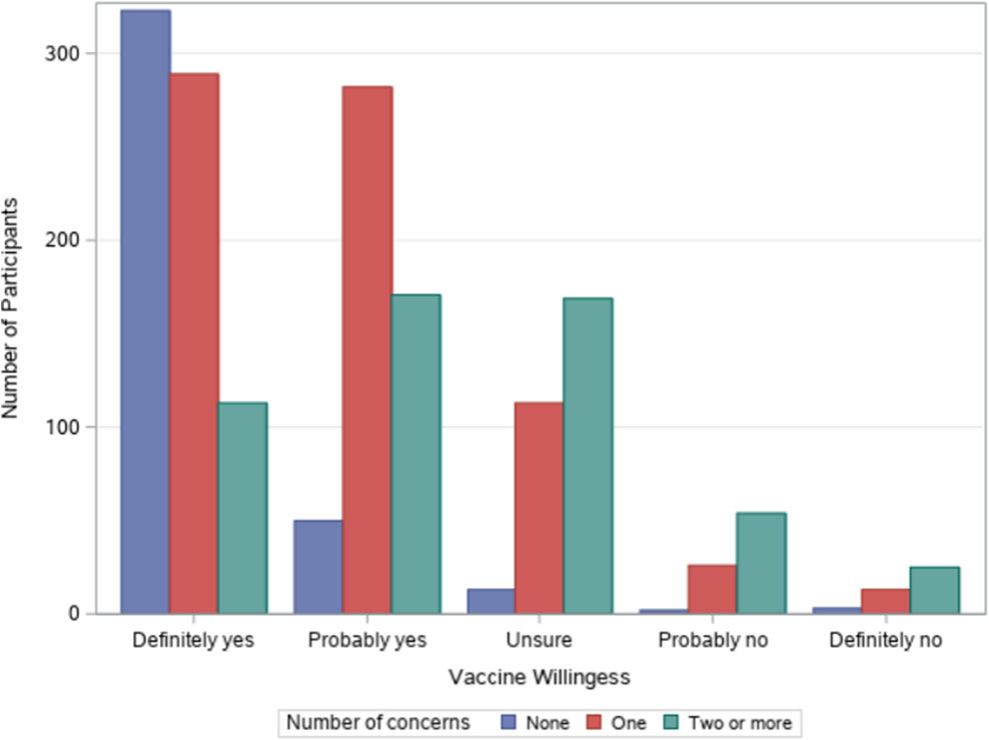
Vaccine willingness by number of concerns (*N* = 1646)

As shown in Table 1, about 24% reported no vaccine concerns. One in three (34.1%) were only concerned about side effects, 6.0% were only concerned about vaccine safety, and 36.4% reported multiple concerns. Figure 1 shows the number of concerns about COVID-19 vaccine by willingness to get the vaccine. Among the 725 participants who said they would definitely get the vaccine, 323 of them (44.6%) said they had no concerns about the vaccine compared to 113 (15.6%) who had two or more concerns. Among the 41 participants who said they definitely would not get the vaccine, 3 of them (7.3%) had no concerns compared with 25 (61.0%) who had two or more concerns.

**Fig. 2:**
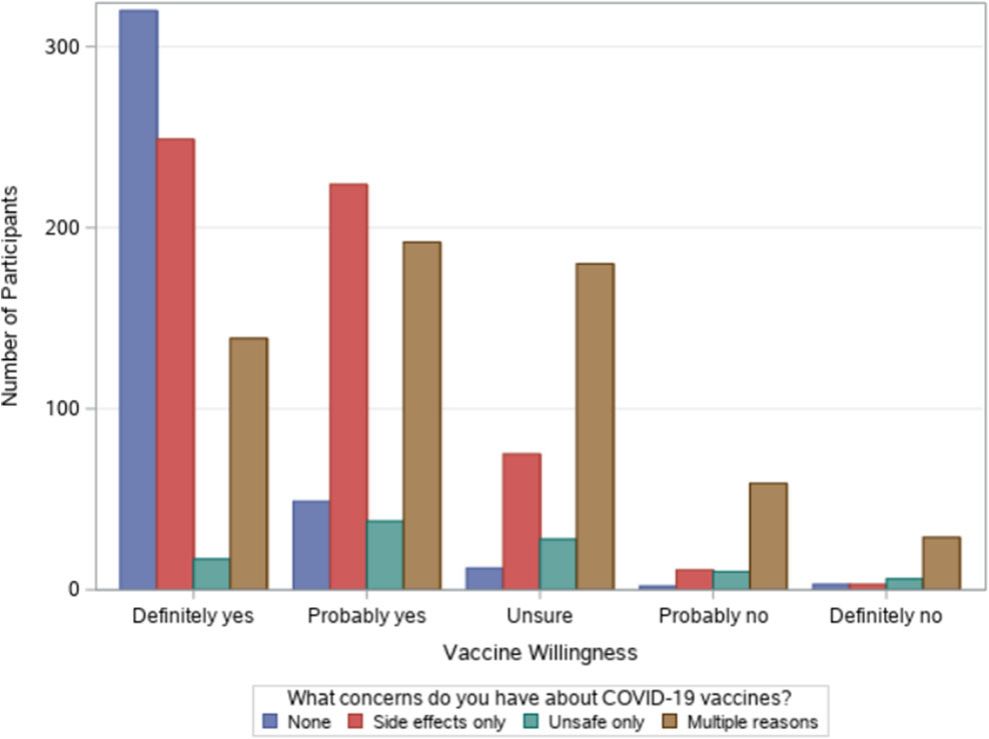
Vaccine willingness by type of concern (*N* = 1646). ^1^Multiple reasons included two or more of the following: (1) I’m concerned about potential side effects; (2) I think COVID-19 vaccine may not be safe; (3) I do not think that COVID-19 is dangerous to my health; (4) I am against vaccination in general; (5) The best way is for nature to take its course; (6) I believe natural or traditional remedies; (7) I’m afraid of injections; (8) religious reasons; and (9) others (please describe)

Regarding the type of concerns (Fig. 2), 249 (34.3%) of those who said they would definitely get the vaccine were concerned only about side effects, 17 (2.3%) were concerned only about the vaccine being unsafe, and 139 (19.2%) were had multiple concerns. Among those who said they definitely would not get the vaccine, 3 (7.3%) were concerned only about side effects, 6 (14.63%) were concerned that only that the vaccine was unsafe, and 29 (70.7%) had multiple concerns.

### Bivariate Analyses

In bivariate analyses (Table 2), cultural group, sex, age, LEP, marital status, income, COVID-19 effect on family income/employment, census regions, and self-reported health status were significantly associated with concerns about the COVID-19 vaccine. On the other hand, race, sexual orientation, nativity, employment status, education, caregiver status, length of SIP order, and COVID-19 severity region were not.

**Table 2:** Concerns about COVID-19 vaccines by participant characteristics (*N* = 1646)

	Unsafe		Side effects		Number of concerns		
	Yes *n* = 544 (%)	No *n* = 1102 (%)	Yes *n* = 1070 (%)	No *n* = 576 (%)	None *n* = 391 (%)	One *n* = 723 (%)	Two or more *n* = 532 (%)
Race							
Asian	532 (33.1)	1075 (66.9)	1050 (65.3)	557 (34.7)	383 (23.8)	705 (43.9)	519 (32.3)
NHPI^1^	12 (30.8)	27 (69.2)	20 (51.3)	19 (48.7)	8 (20.5)	18 (46.2)	13 (33.3)
*P* value	0.864		0.088		0.889		
Cultural group							
Ethnic Chinese^2^	215 (35.2)	396 (64.8)	418 (68.4)	193 (31.6)	127 (20.8)	274 (44.8)	210 (34.4)
Filipino	21 (29.6)	50 (70.4)	60 (84.5)	11 (15.5)	7 (9.86)	40 (56.3)	24 (33.8)
Korean	131 (38.8)	206 (61.1)	213 (63.2)	124 (36.8)	70 (20.8)	159 (47.2)	108 (32.0)
Vietnamese	125 (26.2)	352 (73.8)	282 (59.1)	195 (40.9)	152 (31.9)	190 (39.8)	135 (28.3)
Others	52 (34.7)	98 (65.3)	97 (64.7)	53 (35.3)	35 (23.3)	60 (40.0)	55 (36.7)
*P* value	< .01		< .001		< .001		
Sex							
Female	347 (33.8)	681 (66.2)	715 (69.6)	313 (30.4)	296 (20.0)	470 (45.7)	352 (34.2)
Male	193 (32.1)	408 (67.9)	342 (56.9)	259 (43.1)	182 (30.3)	245 (40.8)	174 (28.9)
Others/decline^3^	4 (23.5)	13 (76.5)	13 (76.5)	4 (23.5)	3 (17.6)	8 (47.1)	6 (35.3)
*P* value	0.558		< .001		< .001		
Sexual orientation							
Heterosexual	497 (33.6)	981 (66.4)	955 (64.6)	523 (35.4)	355 (24.0)	639 (43.2)	484 (32.8)
Not heterosexual	26 (27.4)	69 (72.6)	63 (66.3)	32 (33.7)	22 (23.1)	45 (47.4)	28 (29.5)
Decline to state	19 (27.5)	50 (72.5)	49 (71.0)	20 (29.0)	14 (20.3)	38 (55.1)	17 (24.6)
*P* value	0.279		0.531		0.361		
Age (in years)							
< 30	191 (35.7)	344 (64.3)	364 (68.0)	171 (32.0)	128 (23.9)	220 (41.1)	187 (35.0)
30–39	124 (36.6)	215 (63.4)	230 (67.9)	109 (32.1)	62 (18.3)	157 (46.3)	120 (35.4)
40–49	86 (36.3)	151 (63.7)	158 (66.7)	79 (33.3)	49 (20.7)	107 (45.2)	81 (34.2)
50–59	88 (29.8)	207 (70.2)	190 (64.4)	105 (35.6)	66 (22.4)	140 (47.5)	89 (30.2)
> 60	55 (22.9)	185 (77.1)	128 (53.3)	112 (46.7)	86 (35.8)	99 (41.3)	55 (22.9)
*P* value	< .001		< .001		< .001		
Nativity							
Foreign-born	323 (31.9)	689 (68.1)	649 (64.1)	363 (35.9)	249 (24.6)	445 (44.0)	318 (31.4)
US-born	217 (35.1)	402 (64.9)	411 (66.4)	208 (33.6)	139 (22.5)	272 (43.9)	208 (33.6)
Don’t know	4 (26.7)	11 (73.3)	10 (66.7)	5 (33.3)	3 (20.0)	6 (40.0)	6 (40.0)
*P* value	0.370		0.642		0.785		
LEP^4^							
Yes	102 (30.5)	233 (69.5)	187 (55.8)	184 (44.2)	100 (29.8)	138 (41.2)	97 (29.0)
No	442 (33.7)	869 (66.3)	883 (67.4)	428 (32.6)	291 (22.2)	585 (44.6)	435 (33.2)
*P* value	0.257		< .001		< .05		
Marital status							
Single	196 (34.7)	368 (65.3)	382 (67.7)	182 (32.3)	123 (21.8)	252 (44.7)	189 (33.5)
Married/living with partner	322 (32.6)	666 (67.4)	633 (64.1)	355 (35.9)	235 (23.8)	442 (44.7)	311 (31.5)
Separated/divorced/widowed	22 (26.5)	61 (73.5)	46 (55.4)	37 (44.6)	32 (38.6)	23 (27.7)	28 (33.7)
Declined	4 (36.4)	7 (63.6)	9 (81.8)	2 (18.2)	1 (9.1)	6 (54.5)	4 (36.4)
*P* value	0.477		0.074		< .05		
Employment status							
Full-time	251 (34.1)	484 (65.9)	476 (64.8)	259 (35.2)	165 (22.5)	325 (44.2)	245 (33.3)
Part-time	95 (33.1)	192 (66.9)	184 (64.1)	103 (35.9)	65 (22.7)	135 (47.0)	87 (30.3)
Homemaker	54 (36.0)	96 (64.0)	100 (66.7)	50 (33.3)	33 (22.0)	67 (44.7)	50 (33.3)
Unemployed	65 (30.5)	148 (69.5)	147 (69.0)	66 (31.0)	55 (25.8)	86 (40.4)	72 (33.8)
Retired	39 (27.7)	102 (72.3)	84 (59.6)	57 (40.4)	45 (31.9)	59 (41.8)	37 (26.3)
Others/declined	40 (33.3)	80 (66.7)	79 (65.8)	41 (34.2)	28 (22.3)	51 (42.5)	41 (34.2)
*P* value	0.629		0.598		0.512		
Education							
High school or less	69 (27.9)	178 (72.1)	160 (64.8)	87 (35.2)	57 (23.1)	120 (48.6)	70 (28.3)
Some college or technical school	73 (30.7)	165 (69.3)	147 (61.8)	91 (38.2)	68 (28.6)	101 (42.4)	69 (29.0)
Bachelor’s degree	226 (35.7)	407 (64.3)	422 (66.7)	211 (33.3)	147 (23.2)	262 (41.4)	224 (35.4)
Master’s degree or higher	171 (33.7)	336 (66.3)	325 (64.1)	182 (35.9)	115 (22.7)	229 (45.2)	163 (32.1)
*P* value	0.131		0.565		0.174		
Annual household income in 2019 ($)							
≤ 25,000	81 (28.6)	202 (71.4)	160 (56.5)	123 (43.5)	92 (32.5)	107 (37.8)	84 (29.7)
> 25,000–75,000	171 (10.4)	359 (67.7)	351 (66.2)	179 (33.8)	125 (23.6)	237 (44.7)	168 (31.7)
> 75,000–150,000	137 (33.6)	271 (66.4)	270 (66.2)	138 (33.8)	91 (22.3)	189 (46.3)	128 (31.4)
> 150,000	92 (35.7)	166 (64.4)	166 (64.3)	92 (35.7)	61 (23.6)	106 (41.1)	91 (35.3)
Decline to state	63 (37.7)	104 (62.3)	123 (72.7)	44 (26.3)	22 (13.2)	84 (50.3)	61 (36.5)
*P* value	< .05		< .01		< .001		
Caregiver							
Yes	114 (32.1)	241 (67.9)	234 (65.9)	121 (34.1)	84 (23.7)	156 (43.9)	115 (32.4)
No	430 (33.3)	861 (66.7)	836 (64.8)	455 (35.2)	307 (23.8)	567 (43.9)	417 (32.3)
*P* value	0.671		0.685		0.999		
Length of SIP^5^ order							
No order	29 (31.2)	64 (69.8)	63 (67.7)	30 (32.3)	22 (23.7)	43 (46.2)	28 (30.1)
< 1 month	25 (22.5)	86 (77.5)	67 (60.4)	44 (39.6)	29 (26.1)	59 (53.2)	23 (20.7)
1 to < 2 months	82 (36.1)	143 (63.9)	150 (66.1)	77 (33.9)	54 (23.8)	93 (41.0)	80 (35.2)
2 to < 3 months	72 (32.3)	151 (67.7)	148 (66.4)	75 (33.6)	48 (21.5)	99 (43.4)	76 (34.1)
3 months or longer	289 (33.8)	566 (66.2)	555 (64.9)	300 (35.1)	204 (23.9)	369 (43.1)	282 (33.0)
Don’t know	45 (34.1)	87 (65.9)	85 (64.4)	47 (35.6)	32 (24.2)	58 (43.9)	42 (31.8)
*P* value	0.215		0.892		0.506		
Severity of COVID-19 where you live							
A lot less	48 (32.9)	98 (67.1)	104 (71.2)	42 (28,8)	31 (21.2)	60 (41.1)	55 (37.8)
Somewhat less	104 (30.8)	234 (69.2)	222 (65.7)	116 (34.3)	73 (21.6)	166 (49.1)	99 (29.3)
About the same	144 (34.5)	273 (65.5)	271 (65.0)	146 (35.0)	95 (22.8)	187 (44.8)	135 (32.4)
Somewhat more	159 (31.7)	343 (68.3)	316 (63.0)	186 (37.0)	134 (26.7)	208 (41.4)	160 (31.9)
A lot more	87 (36.9)	149 (63.1)	151 (64.0)	85 (36.0)	57 (24.2)	98 (41.5)	81 (34.3)
*P* value	0.526		0.464		0.337		
COVID-19 effect on family income/employment							
No change	178 (30.3)	409 (69.7)	364 (62.0)	223 (38.0)	157 (26.8)	255 (43.4)	175 (29.8)
Mild	185 (33.4)	369 (66.6)	381 (68.8)	173 (31.2)	111 (20.0)	262 (47.3)	181 (32.7)
Moderate	154 (35.1)	285 (64.9)	287 (65.4)	152 (34.6)	110 (25.1)	175 (39.9)	154 (35.1)
Severe	21 (37.5)	35 (62.5)	31 (55.4)	25 (44.6)	11 (19.6)	28 (50.0)	17 (30.4)
*P* value	0.347		< .05		0.062		
Census region							
Midwest	24 (26.7)	66 (73.3)	64 (71.1)	26 (28.9)	22 (24.4)	40 (44.4)	28 (31.1)
Northeast	57 (40.1)	85 (59.9)	87 (61.3)	55 (38.7)	34 (23.9)	58 (40.9)	50 (35.2)
South	119 (39.0)	185 (61.0)	184 (60.3)	121 (39.7)	76 (24.9)	126 (41.3)	103 (33.8)
West	344 (31.0)	765 (69.0)	735 (66.3)	374 (33.7)	259 (23.3)	499 (45.0)	351 (31.7)
*P* value	< .01		0.108		0.910		
Self-reported health, quintiles							
Quintile 1 (lowest)	120 (34.8)	225 (65.2)	239 (69.3)	106 (30.7)	64 (18.6)	166 (48.1)	115 (33.3)
Quintile 2	93 (35.2)	171 (64.8)	172 (65.2)	92 (34.8)	65 (24.6)	107 (40.5)	92 (34.9)
Quintile 3	116 (35.8)	208 (64.2)	225 (69.4)	99 (30.5)	73 (22.5)	134 (41.4)	117 (36.1)
Quintile 4	109 (31.0)	242 (69.0)	217 (61.8)	134 (38.2)	92 (26.2)	151 (43.0)	108 (30.8)
Quintile 5 (highest)	77 (28.5)	193 (71.5)	162 (60.0)	108 (40.0)	76 (28.2)	120 (44.4)	74 (27.4)
*P* value	0.060		< .05		< .01		

^1^Native Hawaiian and Pacific Islanders

^2^Ethnic Chinese includes mainland Chinese, Hongkonger, Taiwanese, and Huaren

^3^Others: *n* = 7; decline: *n* = 6

^4^English proficiency categorized as limited if speaking or writing or reading English indicated as “some,” “a little,” or “not at all”

^5^*SIP* shelter-in-place

### Multivariable Analyses

In the multivariable models (Table 3), cultural group was associated with vaccine concerns, with Vietnamese significantly less likely to have any concerns about the vaccine compared to ethnic Chinese: adjusted odds ratio (aOR) 0.60 [95% CI 0.43–0.82], *P* < .01. The difference for other cultural groups was not statistically significant relative to ethnic Chinese (reference group). When changing the reference group to Vietnamese in post-hoc analyses, Filipinos and Koreans were significantly more likely than Vietnamese to have any concerns about the vaccine, aOR 3.42 [95% CI 1.48–7.92], *P* < .01) and 1.64 [95% CI 1.13–2.39], *P* =.01), respectively (data not shown).

**Table 3:** Crude and adjusted odds ratios (ORs) and 95% confidence intervals (CIs) for COVID-19 vaccine concerns1

Characteristics	N	Crude OR	Adjusted OR1
Race			
Asian	1607	Reference	N/A
NHPI^2^	39	1.19 (0.54–2.62)	
*P* value		0.66	
Cultural group			
Ethnic Chinese^3^	524	Reference	Reference
Filipino	71	2.38 (1.06–5.31)	2.04 (0.89–4.68)
Korean	337	0.99 (0.71–1.38)	0.97 (0.67–1.41)
Vietnamese	478	0.58 (0.44–0.76)	0.60 (0.44–0.83)
Others/mixed	307	0.85 (0.56–1.31)	0.79 (0.50–1.25)
*P* value		< 0.0001	0.003
Sex			
Female	1028	Reference	Reference
Male	601	0.56 (0.45–0.71)	0.52 (0.40–0.67)
*P* value		< 0.0001	< 0.0001
Sexual orientation			
Heterosexual	1478	Reference	N/A
Non-heterosexual	95	1.03 (0.63–1.68)	
*P* value		0.65	
Age, years			
< 30	535	Reference	Reference
30–39	339	1.42 (1.01–2.00)	2.05 (1.32–3.18)
40–49	237	1.19 (0.82–1.73)	1.72 (1.05–2.82)
50–59	295	1.15 (0.81–1.61)	1.56 (0.98–2.49)
≥ 60	240	0.56 (0.40–0.78)	0.99 (0.61–1.60)
*P* value		< 0.0001	0.002
Nativity			
US-born	1012	Reference	N/A
Foreign-born	619	0.90 (0.71–1.14)	
*P* value		0.64	
LEP^4^			
Yes	335	0.72 (0.55–0.96)	0.81 (0.56–1.17)
No	1311	Reference	Reference
*P* value		0.02	0.25
Marital status			
Single	564	Reference	Reference
Married/living with partner	988	0.91 (0.71–1.16)	0.82 (0.56–1.20)
Separated/divorced/widowed	83	0.44 (0.27–0.72)	0.33 (0.18–0.62)
*P* value		0.01	0.004
Employment status			
Full-time	735	Reference	N/A
Part-time	287	1.00 (0.72–1.39)	
Homemaker	150	1.10 (0.72–1.70)	
Unemployed	213	0.87 (0.61–1.24)	
Retired	141	0.61 (0.41–0.91)	
Others/declined	120	0.90 (0.57–1.42)	
*P* value		0.21	
Education			
≤ High school	247	Reference	N/A
Some college or technical	238	0.73 (0.48–1.10)	
Bachelor’s degree	633	0.96 (0.67–1.36)	
≥ Master’s degree	507	0.99 (0.69–1.42)	
*P* value		0.33	
Household income ($)			
≤ 25,000	283	Reference	Reference
> 25,000–75,000	530	1.49 (1.08–2.05)	1.19 (0.82–1.73)
>75,000–150,000	408	1.60 (1.14–2.25)	1.07 (0.7–1.62)
> 150,000 +	127	1.55 (1.05–2.27)	0.97 (0.59–1.6)
*P* value		0.001	0.10
Length of SIP^5^ order			
No order	93	Reference	N/A
< 1 month	111	1.14 (0.60–2.16)	
1 ≤ 2 months	227	0.94 (0.53–1.66)	
2 ≤ 3 months	223	0.91 (0.51–1.61)	
≥ 3 months	855	0.99 (0.60–1.64)	
*P* value		0.97	
Severity of COVID-19			
A lot less	146	0.94 (0.59–1.49)	N/A
Somewhat less	338	0.96 (0.68–1.36)	
About the same	417	Reference	
Somewhat more	502	1.24 (0.92–1.69)	
A lot more	236	1.08 (0.74–1.58)	
*P* value		0.46	
COVID-19 effect on family income/employment			
No change	587	Reference	Reference
Mild	554	1.46 (1.11–1.93)	1.42 (1.05–1.93)
Moderate	439	1.10 (0.83–1.46)	1.10 (0.79–1.52)
Severe	56	1.66 (0.82–3.38)	2.16 (0.95–4.93)
*P* value		0.04	0.05
Census region			
Midwest	90	0.92 (0.56–1.52)	N/A
Northeast	142	0.94 (0.63–1.42)	
South	305	0.90 (0.67–1.20)	
West	1109	Reference	
*P* value		0.89	
Self-reported health, quintiles			
Quintile 1 (lowest)	345	Reference	Reference
Quintile 2	264	0.68 (0.46–1.01)	0.59 (0.39–0.89)
Quintile 3	324	0.77 (0.53–1.12)	0.69 (0.46–1.02)
Quintile 4	351	0.65 (0.45–0.93)	0.63 (0.43–0.93)
Quintile 5 (highest)	270	0.58 (0.40–0.85)	0.53 (0.36–0.80)
*P* value		0.06	0.03

^1^Any vs. none (reference)

^2^Native Hawaiian and Pacific Islanders

^3^Ethnic Chinese includes mainland Chinese, Hongkonger, Taiwanese, and Huaren

^4^English proficiency categorized as limited if speaking or reading or writing English was indicated as “some,” “a little” or “not at all”

^5^*SIP* shelter-in-place

Age was also associated with having any vaccine concerns: compared to those less than 30 years of age, those 30–39 years of age and those 40–49 years of age were significantly more likely to have any vaccine concerns: aOR 2.03 [95% CI 1.31–3.15], *P* < .01) and 1.69 [95% CI 1.03–2.78], *P* = .04), respectively. All other age groups were not significant relative to those less than 30 years of age (reference group).

Sex was associated with concerns about the vaccine, with males significantly less likely to have any concerns compared to females, with an aOR of 0.52 [95% CI 0.41–0.68], *P* < .01). Self-reported health status was also a significant correlate of having vaccine concerns. Most of those who had higher self-reported health were significantly less likely to have vaccine concerns (a0Rs ranged from 0.53 to 0.58). Marital status was associated with vaccine concerns, with those separated, divorced, or widowed significantly less likely to have any concerns about the vaccine compared to those who were single: aOR 0.33 [95% CI 0.18 – 0.61], *P* < .01). Income and LEP were not associated with having any vaccine concerns in the final model. Finally, respondents reported experiencing mild negative effect on family income/employment due to COVID-19 were more likely to report vaccine concerns when compared to those experiencing no change.

### Open-Ended Response Analysis

A thematic analysis of the 61 open-ended responses revealed five major themes including (1) vaccine trial done too quickly; (2) distrust of government involvement in trial; (3) worry about side/long-term effects; (4) concerns about efficacy and effectiveness; and (5) appropriateness for own body/self. Table 4 shows sample quotes that illustrate each of the theme. Concerns about safety and distrust of how rapidly the vaccine trial was completed were quite salient. Other issues raised included concerns about cost and lack of equitable distribution of the vaccine.

**Table 4:** Themes from open-ended responses^1^

Theme	Quotes
1. Vaccine trial done too quickly	“Afraid vaccine was rushed and not tested well”
	“I am worried that the vaccine will make me sick because it's being rushed and not given enough time for testing”
2. Distrust of government involvement in vaccine trial	“Distrust of the current political administration”
	“Concerned about rollout of vaccine being politically motivated and potentially at the expense of safety”
3. Worry about side/long-term effects	“I’d like to wait a couple years to watch for side effects and efficacy before I feel it may be safe to take the vaccine”
	“Concern about long term effects that may not yet be known”
4. Concern about efficacy and effectiveness	“It may not be effective to prevent COVID”
	“Concerned it will require multiple injections, long-term efficacy”
5. Appropriateness for own body/self	“Current pregnancy - worry vaccine won't be safe during pregnancy”
	“I used to have an egg allergy and most vaccines are made of albumen (egg whites), so I generally have a localized allergic reaction to”
	“I’m not sure that it will work on Pacific Islanders”

^1^There were 61 participants who provided text responses for other concerns that they had regarding COVID-19 vaccines

## Discussion

The findings from this COMPASS national survey are among the first to focus on the AAPI population and concerns related to the COVID-19 vaccine right before the first vaccine became available in December 2020. Overall, 76% of the 1646 respondents reported having at least one or more concerns about the vaccine. The most common concern was side effects (65%). We found several characteristics that reflect subpopulations who expressed more or less concerns with receiving the vaccine. In particular, the subgroups who had significant vaccine concerns included those who were 30–39 and 40–49 age group and females. Respondents who indicated experiencing mild negative impacts from COVID-19 on family income also reported more concerns than those reported no change in income. Those who had less vaccine concerns were those who reported the higher health status compared to those who reported the low health status. In addition, those who were aged 60 and older compared to those who were younger than 30 years old or those who were separated, divorced, or widowed had less vaccine concerns than those who reported being single. In addition, Vietnamese American also reported less concerns when compared to Chinese, Filipino, and Korean Americans.

The COMPASS survey included 62.5% female respondents with 53.1% respondents being between 18 and 39 years of age. Female respondents had significantly more concerns about the vaccine compared to male respondents, which was consistent to findings from a US opinion survey [29]. Since the effects may potentially be additive, these findings may have potential implications for women of child-bearing age though there is limited research on the effects of the COVID-19 vaccine in pregnancy. The Centers for Disease Control and Prevention (CDC) and the Independent Advisory Committee on Immunization Practices (ACIP) recommends that people who are pregnant may choose to be vaccinated and should make an informed decision after discussion with their healthcare provider [30]. Those who are pregnant are at increased risk of severe illness from COVID-19; therefore, a personalized discussion is warranted about their likelihood of exposure and potential risk to them and their fetuses if they are infected [30]. However, there are currently limited data on the safety of the COVID-19 vaccines from animal development and reproductive toxicity studies; additional studies are currently underway in those who are pregnant [30].

The findings that Vietnamese Americans had less concerns about the COVID-19 vaccine are consistent with our finding of more willingness to receive the COVID-19 vaccine in this cultural group (vs. Chinese Americans) (under review) [31]. The previously reported findings were consistent with a global online survey that found that Vietnamese Americans were significantly more willing to receive the COVID-19 vaccine (vs. Chinese Americans) [32]. There is limited research on the potential reasons for these differences as this paper is among the first to demonstrate that AAPI groups are heterogeneous in their concerns about the COVID-19 vaccine. The differences between cultural group could not be explained by the variables examined such as demographics or impacts from COVID-19; thus informative qualitative research will be helpful to elucidate these findings.

One novel finding is that vaccine concern was associated with the experience of mild negative impact of COVID-19 on family income when compared to those experienced no impact. This association has not been reported in prior studies with the English-speaking and/or the general US population. Demographics or other individual factors examined could not explain the association. In bivariate analyses, it appeared that those with mild negative impact on family income tended to experience more concerns side effects. Future research is warranted to understand if and how impacts from COVID-19 are associated with vaccine concern.

Similar to prior research, hesitancy about getting the COVID-19 vaccine had to do with safety [33, 34]. Participants in the current study felt that vaccine trials were done too quickly and had concerns about efficacy. Given how long vaccines had taken in the past, from viral sampling to approval, it was highly optimistic that a COVID-19 vaccine would be developed by the summer of 2021 (Ball, 2020). Thus, to have the vaccines manufactured and FDA approval provided all within the span of a year, even with strong evidence, has worried many [3]. Additionally, the study results suggested that distrust of government involvement in the trial and the politization of the vaccine were quite salient. This has been echoed in the USA and other countries’ data indicating distrust in ministries of health and/or institutions through which vaccine information is delivered influences its acceptance [35]. Finally, some participants had concerns that their own health conditions (e.g., egg allergy) would indicate contraindications with the vaccine [36]. While having concerns about potential side effects of the vaccine and/or additional concerns was common in this study sample, a sizable proportion of respondents who indicated “definitely” or “probably yes” to vaccine willingness also reported concerns about side effects and multiple concerns.

## Limitations

There are several potential limitations. First, this study employed a cross-sectional convenient sample design with participants who self-identified AAPI. Findings may not be generalized to all AAPI. However, major demographic variables were measured and assessed, thus identifying important differences between categories. Nonetheless, this survey is among the first and largest survey targeting AAPI nationally yielding the findings that revealed subgroup differences in vaccine concerns. Future studies should confirm these findings in a more representative sample. These findings are important in informing policy and obtaining resources that require tailoring the different needs of the diverse AAPI population. Second, the study data were collected shortly before FDA authorized the first COVID-19 vaccine when efficacy and side effects of the vaccine remained unclear. As more people get vaccinated and the availability of new empirical data for vaccine safety and effectiveness, willingness and concerns of COVID-19 vaccine will likely be changed. This current study provides an important baseline before vaccine authorization to allow future investigation in changes over time.

## Conclusions

COMPASS is one of the first national surveys targeting a large sample of AAPI to understand vaccination concerns and their correlates. AAPI is a diverse population, and this study revealed differences in vaccine concerns across AAPI groups. AAPI cultural group, age, gender, marital status, and health status are significant correlates of vaccine concerns, while nativity and LEP were not associated with vaccine concerns. The experience of negative impact on family income due to COVID-19 was also found to be associated with vaccine concerns but reasons remain unclear. Findings revealed potential targets for patient education needs. Effective strategies to address various vaccine concerns across subgroups of AAPI will be crucial to ensure equity in vaccination uptake.
